# Genetic modification for enhancing bacterial cellulose production and its applications

**DOI:** 10.1080/21655979.2021.1968989

**Published:** 2021-09-14

**Authors:** Reeta Rani Singhania, Anil Kumar Patel, Mei-Ling Tsai, Chiu-Wen Chen, Cheng Di Dong

**Affiliations:** a Department of Marine Environmental Engineering, National Kaohsiung University of Science and Technology, Kaohsiung City, Taiwan; b Department of Seafood Science, National Kaohsiung University of Science and Technology, Kaohsiung City, Taiwan

**Keywords:** Bacterial cellulose, *komagataeibacter*, nanocellulose, genetic engineering, nanocomposite

## Abstract

Bacterial cellulose (BC) is higher in demand due to its excellent properties which is attributed to its purity and nano size. *Komagataeibacter xylinum* is a model organism where BC production has been studied in detail because of its higher cellulose production capacity. BC production mechanism shows involvement of a series of sequential reactions with enzymes for biosynthesis of cellulose. It is necessary to know the mechanism to understand the involvement of regulatory proteins which could be the probable targets for genetic modification to enhance or regulate yield of BC and to alter BC properties as well. For the industrial production of BC, controlled synthesis is desired so as to save energy, hence genetic manipulation opens up avenues for upregulating or controlling the cellulose synthesis in the bacterium by targeting genes involved in cellulose biosynthesis. In this review article genetic modification has been presented as a tool to introduce desired changes at genetic level resulting in improved yield or properties. There has been a lack of studies on genetic modification for BC production due to limited availability of information on whole genome and genetic toolkits; however, in last few years, the number of studies has been increased on this aspect as whole genome sequencing of several *Komagataeibacter* strains are being done. In this review article, we have presented the mechanisms and the targets for genetic modifications in order to achieve desired changes in the BC production titer as well as its characteristics.

## 1. Introduction

Cellulose from plant-based material is a biopolymer of glucose linked with β,1–4 linkage and is an inexhaustible abundant raw material available for utilization by mankind for their benefits [1–3]. In plant biomass cellulose does not exist in pure form and is often linked with hemicellulose and lignin rigidly, hence its separation is a tedious task [4–6]. Celluloses as a biopolymer have obtained major attention in the last few decades due to its biofuel applications via enzymatic hydrolysis where cellulolytic enzymes play a significant role [7–10]. Celluloses are also present in algae, tunicates and are produced by bacteria as an exopolysaccharide. Biopolymer research advances have demonstrated its potential for a variety of applications, particularly the one which is produced by microorganisms such as nanocellulose produced by bacteria [11]. Bacterial celluloses (BCs) are a naturally occurring unique nanopolymer (with 30–80-nm-wide and 3–4-nm-thick ribbon shaped fibers) which is composed of β-(1-4) linked glucan chains, have attracted attention from all over the World due to its excellent properties attributed to the size [12]. BC is produced in pure form which means it is not associated with lignin or other impurities as in case of plant biomass. Even though nanocellulose can be prepared from plants and algae by various pretreatment methods such as acid and enzyme hydrolysis, the properties of these nanocelluloses vary in terms of crystallinity and size. Chemically, all these celluloses are similar in a way that they are polymers of glucose but they have differences in properties attributed to type of bacteria, production method and size of BC produced. Bacterial celluloses unique properties are highly dependent on bacterial species [13]. BCs are remarkable polysaccharide with its exceptional physicochemical properties *viz*. water absorption capacity, plasticity, porosity, malleability, greater biocompatibility, and biodegradability are 10 times higher in strength than plant cellulose. These unique properties enabled this impressive polysaccharide to be employed for diverse applications [14].

There has been an increased number of publication available on production aspect of BC during last decade mainly on bioprocess development for economic and efficient BC production to take the production to commercial level [11,15–20]. The most known ones among bacteria for cellulose production are *Komagataeibacter* species which were formerly known as *Acetobacter* or *Gluconacetobacter*. This is a gram-negative aerobic bacterium which secretes a large quantity of cellulose as microfibrils along the longitudinal axis of the cell, from a row of synthetic sites [21–23]. There are several genera including *Komagataeibacter, Rhizobium, Enterobacter, Burkholderia, Klebsiella, Escherichia, Erwinia chrysanthemi, Agrobacterium*, and *Sarcina*, etc., which are known for bacterial cellulose production, though the production yield varies significantly [12,24–26]. For BC production researchers have employed various carbon sources ranging from glucose, fructose, other defined sources to undefined sources such as fruit pulp, fruit residues, cellulosic waste, textiles waste, tea extract, tobacco extract, etc. and all of them have proven successful [15,27–30]. Most of these studies are on static culture condition. Efforts for producing BC nanocomposites by coculturing of two different bacterial cultures producing different polysaccharides were also analyzed to have synthesized nanomaterial with improved water holding capacity [31]. Thus, bioprocess have been explored for BC production enhancement as well as its improved properties; however, commercial production is still a challenge.

A stable engineering of *Komagataeibacter* strain is utmost necessity for commercial production of bacterial cellulose. Genetic engineering allows modification of the genetic material of *Komagataeibacter* to decrease the risk of harmful/nondesired mutations, improve cellulose production, improve/altered properties of cellulose such as mechanical properties, porosity, crystallinity as suitable for specific applications. Along with above advantages there are some foreseen challenges also that needs to be addressed. Problems have been faced for transformation of few *Komagataeibacter* strains. Few researchers reported inability to transform *K. hansenii* ATCC 58532 even by using electroporation and the reason could be lack of whole genome sequence information and unavailability of genetic toolkit for genetic engineering of cellulose producing bacteria [32,33]. Genetic modification may not always give positive impact on yield as well as properties of cellulose, the reason being the complicated regulatory process where each gene may express the protein having more than one function. Recently, genetic modifications have been geared up due to genome sequencing of several BC producing strains and availability of toolkits for genetic engineering [34,35].

Bacterial cellulose is being considered as an excellent biomaterial for various applications due to which there is an increased demand, however the production efficiencies are still limited. For industrial production of bacterial cellulose significant enhancement in production efficiencies is required. We have tried to address the issue by genetic modification by which potential genes can be targeted for improved production either by blocking genes responsible for synthesis of side metabolic products or by overexpressing the genes involved in biosynthesis of cellulose. There has been a lack of studies on genetic modification for BC production due to limited availability of information on whole genome and genetic toolkits; however, in last few years, whole genome sequencing of several *Komagataeibacter* strains are being done which has resulted in renewed interest in this aspect.

## 2. Mechanism of bacterial cellulose production in *Komagataeibacter xylinus*

*Komagataeibacter xylinus* formerly known as *Gluconobacter/Acetobacter xylinus* is a model bacterium for studying cellulose production. Bacterial cellulose has excellent properties such as high malleability, water retention capacity, high strength, high elasticity, etc. Biosynthesis of BC is a specifically regulated multistep pathway that follows a defined route which include several numbers of both singular enzymes as well as sets of regulatory proteins [36].

**Figure 1.**
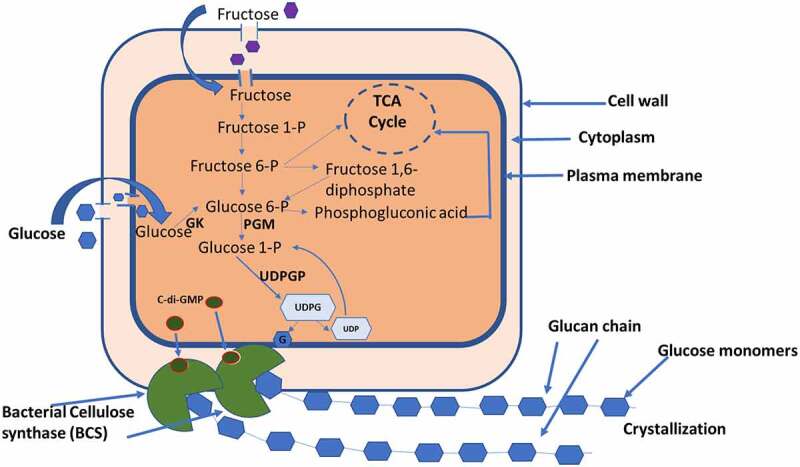
Mechanism of cellulose biosynthesis in *Komagataeibacter xylinus.*

*Komagataeibacter* produces highly ordered cellulose which is synthesized in the periplasmic space by the catalytic activities of a set of enzymes including glucokinase, phosphoglucomutase, UDP-glucose phosphorylase, and cellulose synthase. The pathway to cellulose from the substrate glucose as presented in Figure 1 involves a series of the reactions in which the first step being conversion of glucose into glucose-6-phosphate by enzyme glucokinase. It is followed by conversion of glucose 6-phosphate to glucose-1-phosphate by the enzyme phosphoglucomutase. In the next step of the reaction, glucose-1-phosphate is converted to UDP-glucose in the presence of UTP and the enzyme UDPG pyrophosphorylase. The UDP-glucose is the intermediate sugar nucleotide precursor in *K. xylinum* for cellulose synthesis. Finally, cellulose synthase transfers glucosyl residues from UDP-glucose to the nascent β-d-1,4-glucan chain. It is subsequently polymerized into BC through bacterial cellulose synthase complex [35]. Cellulose synthase is the most important enzyme for cellulose biogenesis as it is the only unique enzyme related to this process and is located in the cytoplasmic membrane. In *A. xylinum*, activation of cellulose synthase is mediated by c-di-GMP (bis-(3′,5′)-cyclic di-guanosinemono-phosphate), which binds to the PilZ domain of the BcsA subunit. Cellulose synthase gets activated allosterically at the posttranslational stage [37–39]. In catalyzing the cellulose biogenesis, the c-di-GMP specifically enhanced the reaction. Diguanylate cyclases (DGCs) are responsible to control the cellular level of c-di-GMP and c-di-GMP-specific phosphodiesterases (PDEs) [40,41].

GK = Glucokinase, PGM = Phosphoglucomutase, UDP = Uridyl diphosphate, UDPG = Uridyl diphosphate glucose, UDPGP = UDPG phosphorylase, C-di-GMP = cyclic diguanylic acid

Two types of operons of cellulose synthase are there, type 1 and type 2 in bacterial genome which encodes bacterial cellulose proteins [42]. The type I bcsI operon comprises following four genes as, bcsAI, bcsBI, bcsCI, and bcsDI. The type 1 operon is flanked by accessory genes (cmcAx, ccpAx and bglAx), which modulate biosynthesis of cellulose process by complementing the bcs operon in polymerization of glucan chains, fibril packaging, and cellulose crystallization [24,43,44].

The type II cellulase synthase operon (bcsII) synthesizes acylated cellulose due to the presence of an acyltransferase gene within operon [42]. Expression of these operons are constitutive; however, the expression fluctuates depending on the growth and environmental conditions [45–47].

The glucose residues are added to the nonreducing ends of the glucan chain and the reducing ends being nascent polymer chains, situated away from the cells. By polymerizing glucose through cellulose synthase and assembling 1,4-glucan chains into the intermediate length of glucan chains which are synthesized by H-linkage into ribbons of <100 nm width at the bacterial cell’s surface [48,49]. These ultra-thin three-dimensional networks of ribbon is called as pellicle [48,49].

The mechanism of cellulose biosynthesis has been studied in *K. xylinum* about three decades back [21]. Biosynthesis of cellulose is growth dependent and is independent of carbon source employed as cellulose biosynthesis needs glucose molecules for cellulose production; hence, glucose is the dead end for cellulose synthesis. A number of carbon source have been employed for cellulose production and the suitability of carbon source can be understood in terms of two metabolic pathways in this bacterium, the pentose phosphate pathway for carbohydrate oxidation and the citric acid cycle for the oxidation of organic acids and related compounds [50,51]. This bacterium lacks phosphofructose kinase, which is required for glycolysis; hence, it cannot metabolize glucose anaerobically [52].

In *K. xylinum*, the unusual regulation of the pyruvate phosphate di-kinase and oxaloacetate decarboxylase, causes gluconeogenesis to occur from oxaloacetate via pyruvate. Phosphorylation of exogenous hexoses generates hexose phosphate in the metabolic pool of the organism where cellulose synthesizes directly whereas, via the pentose cycle and the gluconeogenic pathway, cellulose synthesizes indirectly. Direct synthesis implies that it does not necessarily include intermediary cleavage of the carbon skeleton of the hexose moiety. The flow of hexose phosphate carbon toward cellulose or through the pentose cycle appears to be regulated by an energy-linked control mechanism. ATP-sensitive NAD-linked glucose-6-phosphate dehydrogenase is the crossover point. One of the two glucose-6-phosphate dehydrogenases operative in *K. xylinum*, is inhibited by ATP. Cellulose production in *K. xylinum* does not depend on net protein synthesis, though, it is conditional on concurrent oxidation processes [21]. It was evident when washed cells in absence of nitrogen source continued to produce cellulose. BC production in *K. rhaeticus* ENS9a in nitrogen-free medium was reported which led to find the gene annotations related to nitrogen fixation. Genes homologous to nifHDK, which forms the main nitrogenase subunits in *G. diazotrophicus* was not found. Use of PBS treated *K. rhaeticus* ENS9a cells in the test eliminates possible nitrogen contamination, indicating that the strain might contain different set of nitrogen fixation and regulatory genes [33].

A set of four enzymes are required to drive cellulose synthesis in the extract from UDP glucose namely, glucokinase, phosphoglucomutase, UDP-glucose phosphorylase, and cellulose synthase reaction along with other proteins and regulators. These could be the probable targets for genetic modifications for enhancements or controlled production of cellulose in *K. xylinum*.

## 3. Genetic modification for enhanced/regulated BC production

Major challenge in higher-scale production of BC from *Komagataeibacter* strain is the lack of cost effectiveness due to the low productivity. One of the major drawbacks is that there is huge variation in the nutritional requirements as well as production efficiency of the various *Komagataeibacter* strains and the formation of unstable Cel-mutants (non-BC producing mutants) spontaneously in agitated cultures which leads to consumption of nutrition for growth and multiplication of cells without cellulose production [12]. There are many reports where static and agitated conditions have been analyzed for cellulose production by *Komagataeibacter* as well as other bacteria such as *Rhodococcus* sp. where static culture invariably gave higher yield as compared to agitated cultures [53,54]; however, few reports of higher BC yield with agitated culture have been reported as compared to static culture [55]. It is possible for Cel-mutants to regain their cellulose-producing ability without shaking under optimal conditions due to reversible phenotypic switch [56]. However, Cel-mutants due to genotypic conversions are irreversible as it may be due to mutation in genes involved in biosynthesis of cellulose, where it is not possible to revert back to Cel+mutants (cellulose producing strain) without any further genetic modification. A bcsA gene was altered with an insertion sequence element and responsible for generating the Cel-mutant strain [57]. It was further demonstrated bcsA gene DNA sequence was engineered so as to reduce the efficiency of getting common insertion sequence inserted and disrupt its function. This genetically modified strain retained its bcsA gene and displayed 1.7 times increase in cellulose production in comparison to natural unmodified strain without displaying any changes in chemical and physical properties [57].

**Figure 2.**
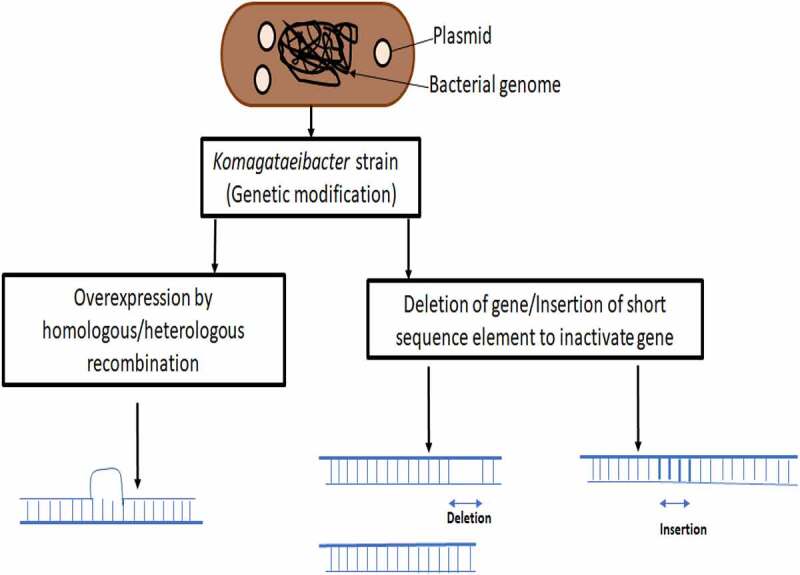
Genetic modification: A general scheme in *K. xylinus.*

Genetic engineering is a useful tool which has been very well explored for bacterial cellulose production, which can vary from overexpressing cellulose, modifying it chemically at genetic level itself to produce bio-composites or to regulate its production. Figure 2 represents a general scheme of genetic modification. Homologous recombination, heterologous gene expression and novel techniques like CRISPR have been employed by researchers to modify genome of BC producing bacteria and recent sequencing of genomes of few *Komagataeibacter* strains have given a required boost to this research direction, otherwise majority of the research was based on isolating novel BC producing bacteria, bioprocess development, bioreactor designing, etc. An account of various kind of modifications at genetic level has been presented in Table 1.

**Table 1.** Genetic modifications and adopted strategies for improved cellulose bioprocess

Bacteria	Media/carbon source and yield	Genetic modification	Objective	Strategy and purpose of genetic modification	References
*Komagataeibacter rhaeticus*	Low nitrogen medium, Rod-like cellulose ~2 µm in length	Targeted UGPase gene, as it catalyzes the production of UDP-glucose critical for cellulose synthesis	To achieve control over cellulose production	sRNA-mediated knockdown of UGPase to inhibit production of UDP glucose	[33]
*K. xylinum* DSM 2325	Glucose in a complex medium, 3.15 g/L BC	Heterologous overexpression of glucose 6-phosphate isomerase *pgi* gene from *E Coli*	To enhance BC production	Integrategration in genome and overexpression of heterologous gene *pgi*	[35]
*Glucanoacetobacter xylinus*	Glucose, 1.38 g/L BC production	Knockout of glucose dehydrogenase gene	To enhance BC production	By homologous recombination of defected GDH gene so as to reduce gluconic acid formation	[70]
*Gluconobacter xylinus*	Glucose, BC production 4.3 g/L and glucose conversion 184.7 mg/g	Heterogeneous expression of the Vitreoscilla hemoglobin (VHb)-encoding gene vgb,	Study oxygen tension effect on BC production	Heterogeneous expression of VHb gene and plasmid pBla-VHb-122 enhanced BC yield in lower oxygen tension	[58]
*K. hansenii* ATCC 23769	Glucose and cellulose	bglxA and cmcAx	Enhanced cellulose production	Transglycosylation as well as hydrolytic activity of betaglucosidase regulate glucose and other oligos concentration to regulate expression of genes	[78]
*K. xylinus* PBR2001	Glucose, 1.7-fold higher BC production, 4.1 g/L	gdh knock down	To enhance BC production	disruption of the gene encoding putative PQQ-GDH	[68]
*K. xylinus* AY201	Glucose	crdS gene introduction and expression to simultaneous synthesize cellulose/curdlan	To synthesize more porous nanocomposites curdlan/cellulose	crdS gene from *Agrobacterium sp* introduced in *K. xylinus* via expression in plasmid for nanocomposites synthesis curdlan/cellulose	[82]
*K. xylinum*	Glucose	An operon of three genes (NAG5, AGM1 and UAP1) from the yeast *Candida albicans* introduced into *K. xylinus*	To reduce crystallinity and increase biodegradability	NAG5, AGM1, and UAP1 introduced into *K xylinum* to synthesize chimeric polymer with enhanced biodegradability	[83]

There are several target sites for improved cellulose production by *K xylinus*. Recently Liu et al. heterologously expressed the *Vitreoscilla hemoglobin* (VHb)-encoding gene vgb, in *Gluconobacter xylinus* via the pBla-VHb-122 plasmid. This gene was extensively applied to improve viability of cell during hypoxia. *G. xylinus* with vgb gene (*G xylinus*-vgb+) could produce 26.5 and 58.6% enhanced cellulose at oxygen tensions of 10 and 15% when compared to *G. xylinus*. The maximum BC yielded was 4.3 g/L with a glucose conversion rate of 184.7 mg/g was obtained by modified strain at 15% oxygen tension. *G xylinus*-vgb+ performs better in hypoxia condition and behaves as regular *G. xylinus* under aerobic condition. Hence, it is proven that oxygen plays a significant role in biosynthesis of cellulose in *G. xyllinus* [58]. DGC and PDEA are the two enzymes with opposing actions which regulates the BC synthesis in *Komagataeibacter* strains by controlling the cyclic diguanylic acid (c-di-GMP) concentration in the cell. DGC catalyzes the formation of c-di-GMP, which regulates BC synthesis positively by specifically activating the cellulose synthase [59]. Hence, it was expected that by disruption of dgc1 the BC production will decrease, however the dgcl disrupted mutants produced BC in similar amounts as that of wild-type strain in shaking as well as static culture. Also, the wild-type strain was growing better than engineered strain. Despite the fact that production of c-di-GMP was crucial for stimulating cellulose synthase, deletion of the c-di-GMP synthesizing gene (dgc1) did not preclude BC biosynthesis [60].

Cellulose biosynthesis in *Komagataeibacter* strains, involves series of enzymes and regulatory proteins along with other substances. Endo-β-1,4-glucanase (CMCase) exhibiting celllulose hydrolytic activity was shown to be critical for cellulose production which activates BC production [61]. In a study about 20% increase in BC productivity was reported after cmcAx overexpression in *Komagataeibacter hansenii*. Even on addition of CMCase in the medium at 1.5 mg/L increased BC production. This indicates CMCase hydrolytic activity may have exerted a regulatory effect on BC production. In *K. xylinus* DSM 2325 two cellulase genes were found bglxA and cmcax which are responsible for cellulose degradation [35] and can be thought to play a role in reducing the BC yield which is not so. However, β-glucosidase are also known to exhibit transglycosylation activity which suggests its role in regulating the glucose and some cello-oligosaccharides concentration, which are possible starting material for the cellulose production, and/or involve in regulating the expression of other important genes [61–65].

*Gluconobacter* genus can utilize various carbon source such as fructose, glucose, sucrose, galactose, xylose, mannose, etc. for producing bacterial cellulose [66,67]. For cellulose-producing bacteria, when disaccharides, such as sucrose and maltose are used as a carbon source, then the disaccharides are hydrolyzed into monosaccharides such as glucose and fructose to gear up biosynthesis of BC. BC production via these carbon sources usually leads to lower conversion efficiencies which often leads to higher cost of bacterial cellulose production. There are many reports on using glucose as carbon source, the reason being its cost effectiveness in comparison to other carbon source but the major limitation of growing *G. xylinus* with glucose is the pyrroloquinoline quinone (PQQ) cofactor-dependent glucose dehydrogenase (GDH) which is located in the cell membrane and is responsible for conversion of glucose to gluconic acid [68,69]. Most of the *G. xylinus* producing higher BC in glucose medium have low GDH activity. Hence it gives an insight that, by eliminating GDH activity, BC pellicle production increases which may lead to economic feasibility [69]. By disrupting membrane bound PQQ-dependent glucose dehydrogenase (GDH) encoding gene of *G. xylinus* via homology recombination a GDH deficient mutant of *G. xylinus* was obtained which could very well utilize glucose to produce BC without generating gluconic acid. About 40% increase in BC production was reported compared to the wild strain [70]. The pH of the medium drops due to oxidation of glucose into gluconic acid which leads to a rapid reduction in concentration of glucose. Hence, it would be important to strategize the reduction of the gluconic acid production which is the main side-product from glucose as carbon source. For materializing this the mutants (*K. xylinus* BPR2001 GD-1) with glucose dehydrogenase gene (gdh) knock-out were constructed [68]. *K. xylinus* BPR2001 GD-1 were GDH-deficient mutants when compared to the wild type strains, produced two times higher BC. It was also found that the *K. xylinus* BPR2001 GD-1 produced 5.0 g/L BC on enzymatically hydrolyzed potato pulp and 7 g/L with addition of ethanol. There are several reports where ethanol and citric acid addition has enhanced the BC production by reducing the main by-product of the citric acid cycle (TCA cycle) [71]. The ethanol supplementation causes an excessive flow of G6P as G6PDH gets inhibited as the ATP spikes and cellulose biosynthesis occurs when the metabolic flow enters the node of G6P. Phosphofructokinase along with pyruvate kinase (PK) activities decreases and metabolic flux gets balanced between the EMP pathway and the TCA cycle so as to minimize the byproducts [71,72].

Conversion of glucose to gluconic acid not only hampers the conversion of glucose to bacterial cellulose but also reduces the pH of the medium significantly which is also deleterious for BC production by bacteria [73,74]. It would be highly interesting to have bacteria which can produce cellulose at low pH. *Komagataeibacter medellinensis* ID13488 is one of recently reported bacteria which is capable to synthesize crystalline BC under highly acidic conditions during growth. This ability makes it a potential candidate for industrial BC production utilizing acidic residues such as the wastes which is generated during cider production [46]. The genomic sequence of the strain *K. medellinensis* ID13488 was reported which shows the difference with non-BC producing strain NBRC 3288 which belongs to the same species. The most significant difference lies in the plasmid content and the genetic makeup of the two operons namely bcs1 and bcs2. The four independent BCS operons exists in the *K. medellinensis* ID13488 genome. The biosynthesis of type I cellulose in the above strain occurs by the operon bcs1 encoding all the required putative protein products necessary for the synthesis of BC. At pH 3.6, this operon got transcribed which is defined as BC production conditions.

Controlled cellulose production is desired for commercialization of the process by bacteria. Genetic engineering can serve as an excellent tool to achieve control over cellulose production by *Acetobacteraceae*. Wild-type bacteria produce cellulose constitutively which imparts a high metabolic cost in industrial production process. In well aerated conditions it leads to formation of noncellulose producing mutants [75]. Hence, it is desirable to prevent cellulose synthesis when it is not required so as to control/regulate multiplication of mutants. Control is also required to control the density of the microfibers of cellulose to avoid the macro size which may change the entire properties compared to nanocellulose [33]. For this, UDPGPase gene has been knockdown by using sRNA that inhibited the synthesis Uridyl diphosphate glucose acting as precursors for cellulose synthesis [23,33].

Studies predicted glucose 6-phosphate isomerase (pgi) and phosphogluconate dehydrogenase (gnd) genes as novel targets of overexpressions for the increased BC synthesis as they showed positive correlation with BC production during random sampling for the total number of 16 reactions from glycolysis and pentose phosphate pathway [35,76]. *K. xylinus* strains were engineered which individually over expressed pgi and gnd genes either from *E. coli* or *Corynebacterium glutamicum. K. xylinum* strain overexpressing the *pgi* gene from *E. coli* produced BC of 3.15 g/L during fermentation in a complex medium with glucose as carbon source which was 115.8% higher as compared to 1.46 g/L BC obtained from the unmodified strain. Data generated from genome sequence could be useful information to enable metabolic engineering of *K. xylinus* for the improved BC production [35]. KxyMBEL1810 was genetically modified version of *K. xylinum* overexpressing two target genes. These results demonstrated that the *pgi* and *gnd* are the two reliable gene having overexpression targets, from glycolysis and PP pathway generated in KxyMBEL1810 which caused enhancing effects on the BC production.

## 4. Genetic modification for altered characteristics of BC

Physicochemical properties of cellulose are influenced by the size and shape of cellulose fibers. Genetic engineering could be served as an excellent tool for modifying bacterial cellulose during production by heterologous expression of enzymes itself. Bacterial cellulose pellicle produced by *Komagataeibacter xylinus* is one of the best biobased materials with remarkable physicochemical properties having a unique super network structure for a wide range of tissue-engineering and medical applications. Still, it is required to modify them to obtain suitable materials for biomedical use with satisfactory biodegradability, mechanical strength, and bioactivity. Though the genetic modification in *Komagataeibacter* mainly focused on improved BC productivity, it has also been investigated for altering the properties of BC to have improved characteristics for specific applications.

To improve BC properties for medical application *K. hansenii* cells were genetically engineered to influence bacterial movement or bacterial morphology. Bacterial motility has been regarded as the complex phenomenon exerting influence on biofilm formation. Flagella helps bacteria to move and has been considered as an essential organelle involved in biofilm formation in its initial stage [77]. Authors claim that for the first time *K. hansenii* ATCC 23769 overexpressed *mot*A and *mot*B gene which displayed elongated cell type as well as increased motility and productivity. It was found that the

cellulose produced consists of thicker ribbons arranged in looser network when compared to wild-type strain. Hence BC membranes produced were highly improved [79]. The mutant-derived BC appeared to be very promising as a support for chondrogenic cells propagation and promoted their chondrogenic-like behavior [80]. The same group tested if the reduced motility in *K. hansenii* ATCC 53582 could produce cellulose pellicle with increased fiber density, hence motility related genes were disrupted by homologous recombination. SEM imaging revealed membranes with significant reduction in fiber diameter and increased network density [81].

There have been several interesting modifications in the BC characteristics majorly due to introduction of another polymer at genetic level giving rise to biosynthesis of biocomposites as the end product by bacteria. For example, the crdS gene from *Agrobacterium spp* ATCC 31749 for curdlan synthesis was introduced into *G. xylinus* AY201 from a plasmid via expression which led to UDPG being polymerized intracellularly to secrete cellulose along with curdlan parallelly in modified cells [82]. Authors aimed to develop a gene-transformation route for the production of bacterial nanocomposites cellulose/curdlan (β-1,3-glucan) by separate but simultaneous in vivo synthesis of both polysaccharides which was successful. The obtained biocomposites were characterized, and their properties were compared with those of normal bacterial cellulose pellicles, indicated that curdlan mixed with the cellulose nanofibers at the nanoscale without disruption of the nanofiber network structure in the pellicle [82].

Yadav et al. has engineered cells to add *N*-acetylglucosamine in cellulose fibers which reduced its immunogenicity and increased biodegradability [83]. Bacterial cellulose can also be functionalized with purified proteins which allows for a wider range of materials (bio-composites) to be engineered for various medical applications. It could be an alternative to the previous strategy. In order to enable external control of gene expression, Florea et al. [33] developed a genetic toolkit including protocols, flexible plasmids, robust promoters, reporter proteins, and inducible constructs.

Cellulose resistivity to in vivo degradation limited its applications in reconstruction of tissues or other biomedical applications. Yadav et al. addressed this issue by introducing an operon from *Candida albicans* into *K. xylinus* with three genes such as AGM1, NAG5 and UAP1. These genes were expressed in *K. xylinus* and encoded a metabolic pathway in which *N*-acetylglucosamine (GlcNAc), a monomer of chitin is converted inside the cell into UDP-GlcNAc. A chimeric polymer derived from these monomers by cellulose synthase contained glucose and GlcNAc. High GlcNAc content and lower crystallinity make this biocomposite a multifunctional bioengineered polymer susceptible to lysozyme as chitin can be degraded by lysozyme widespread in the human body and can naturally get degraded when implanted in vivo [83].

## 5. Applications

BC’s high purity, hydrophilicity, chirality, structure forming potential and biocompatibility offers a wide range of special applications, e.g. as a dietary fiber, as food matrix (nata de coco), as an acoustic or filter membrane, as ultra-strength paper, as tissue grafting for wound dressing, biotechnological applications for enzyme and cell immobilization, etc. [84,85]. Table 2 presents an account of various applications of BC in different industries. Its versatility, as well as the fact that it can be made in various shapes and textures, essentially gives BC a wide variety of food applications. A culture medium source such as fruit syrup can be used to grow bacteria that produce cellulose with the characteristic flavor and pigment of the fruit [11,86].

**Table 2.** Applications of BC in various industries based on its specific properties

Industry	Application	Properties of BC enabling application
Cosmeceuticals	Face masks, stabilizer of emulsions like conditioner, cream, tonics, nail polishes, make-up pads	Moisturizer retention capacity
Mining and refinery	Sponges to collect leaking oils, material for absorbing noneco-friendly discharges like toxins	Water holding/retention capacity
Textile industry	Tents and camping equipment, sports-related nonwoven clothing	High mechanical strength
Sewage treatment	Recycling of minerals and oils, filtration of sewage, and water purification	BC as potential material for membrane synthesis
Communications	Diaphragms for microphones and stereo headphones	High mechanical strength
Food industry	Diet food and drink with low calories, edible cellulose (nata de coco)	Low digestible sugar content, ability to reduce the cholesterol level
Paper industry	Artificial replacement of wood, Flexible/durable and high strength paper, special papers suitable for currency printing	Extremely small clusters of cellulose microfibrils with higher filler content
Medicine/ biomedical	Temporary artificial skin for burns and ulcers, dental implant components; antimicrobial wound dressing, nanofilm, drug delivery, drug excipient.	Ability to absorb exudates during the inflammatory phase
Laboratories	Protein immobilization, chromatographic techniques, tissue culture medium	Water-retention capacity
Electronics	Optoelectronics materials (liquid crystal displays)	Antibacterial properties
Energy	Membrane fuel cell (palladium), catalyst precursor	-

BC has been investigated as a binder in papers, and because it consists of extremely small clusters of cellulose microfibrils, this property greatly adds to strength and durability of pulp when integrated into paper. BC when combined with wood cellulose papers, then the resulting biomaterial exhibited increased barrier properties [87]. BC has excellent mechanical properties, which makes it ideal for the restoration of damaged paper documents, where its surface lining does not impair document legibility [88–90].

BC has several applications in human and veterinary medicine due to its reticulated fine fiber network with coating, binding, thickening and suspending characteristics. Besides providing excellent mechanical properties, BC’s 3D nanoscaled network structure allows it to serve as a natural scaffold for a variety of tissue regenerations [91]. Among its many advantages, BC is biocompatible, conformable, elastic, transparent, is able to maintain a moist wound environment, and accounts for absorbing exudates during the inflammatory phase [92]. This biomaterial has been employed in an array of exciting biomedical applications including wound dressings, artificial skin, scaffolds for tissue engineering, vascular grafts, artificial blood vessels, dental implants, and medical pads [93,94]. Potential applicability of BC has been explored in drug delivery systems by Müller et al. [95] using serum albumin as the model drug. It was observed that the samples which were freeze-dried had higher albumin uptake capacity when compared to native BC, possibly because the fiber network is altered during freeze drying. It is possible to preserve the integrity and biological activity of proteins during the loading and releasing process.

Among others, Hu et al. [96] reviewed the benefits and different uses of functional nanomaterials based on BC, with a special focus on the use for sensors, photocatalysts, optoelectronics, and magnetically responsive membranes. Additionally, Shah and colleagues [93] presented the significant applications of BC composites in biomedical products, electrical devices, conducting materials, separation and waste purification, and industrial applications of nanocomposites with high mechanical strength. Environmental applications for BC as nanofiber composite adsorbent for highly efficient removal of bisphenol A gives a new insight [97]. BC also finds applications as catalyst precursor of the microbial fuel cell cathode and was found better than platinum [98]. Hence, BC has been emerged as an excellent bionanomaterial for various applications in almost every field due to its versatile characteristics.

## 6. Future perspectives

In last decade there were majority of research articles published on bacterial cellulose mainly focused on isolation, bioprocess development for its production as well as on its applications. It has been very well documented that there is a great demand for bacterial cellulose due to its extremely fascinating properties, which allowed its applications in diverse fields starting from food industries, cosmeceuticals, paper industry, biomedicals to biosensors. Further efforts are necessary to improve its production ability so as to make this biotechnological material commercially viable, economically feasible and a competitive product. The strict aerobic nature as well as requirement of static conditions in major cases, for pellicle formation, are the two contradictory paths which requires major interventions from biochemical engineers to design a process for mass transfer without agitation so as to achieve maximum yield of BC. Genetic intervention could play a significant role in enhancing BC production for which it is necessary to understand the mechanism of its production in bacteria. Till date *Komagataeibacter* are known as the best producers, hence, has been studied as a model organism for BC production. Several enzymes and regulatory proteins are required for BC synthesis and ‘up and down’ regulation of these proteins could improve the yield or properties as well, to be employed for specific applications. Availability of genome sequences of BC producing bacteria in last few years have provided a boost to research in this direction and further progress is expected with availability of genetic toolkits for genetic modifications.

## 7. Conclusions

Bacterial cellulose is considered as an excellent biomaterial due to its unique properties such as high tensile strength, water retention capacity, excellent malleability, etc. *Komagaetaeibactor* strains have been most explored as are excellent source of BC with higher production capability. Industrial production of BC is still limited due to low yield which can be improved by blocking side products metabolism. It can be possible by genetic modifications where genes are targeted for either overexpression or deletion to have improved yield of BC. It is a complicated pathway as multiple number of genes and regulatory proteins are involved and hence mechanism of biosynthesis needs to be understood. Whole genome information and availability of genetic toolkits have enabled researchers worldwide to target one or the other gene so as to introduce desired trait in BC. Genetic engineering is a promising tool enabling to introduce desired changes in the product.
